# Notes on Medical Matters in Paris

**Published:** 1847-01

**Authors:** David W. Yandell

**Affiliations:** Louisville, Ky.


### THE WESTERN JOURNAL OF MEDICINE AND SURGERY.

JANUARY, 1847.

ART. I.—*Notes on Medical Matters in Paris.* By DAVID W. YANDELL, M.D., of Louisville, Ky.

After a sojourn of several weeks in the emporium of medical science I do not find myself in possession of a sufficient amount of professional matter to form a communication for the Journal such as I had hoped to make; but having come under a promise to furnish one for each number, the following is submitted, such as it is. I begin with a report of a clinical lecture on *Orchitis*, by Professor Velpeau, at the Charity Hospital.

Of the twenty-four examples of orchitis which you have seen in the hospital this year, said M. V., eighteen commenced in the urethra; the origin of the six others has remained doubtful. It is generally admitted that acute orchitis com prises two distinct varieties, the first of which has its origin in a lesion of the urethra; the second is produced by external violence. There is in our opinion a third class intermediate between the two others, if we may use the expression, which is not so generally recognized among practitioners. This variety arises neither from a lesion of the urethra, nor from any external violence, but it is caused, we believe, and the patients themselves sustain the opinion, by a violent effort or strain.

It is generally the case when a man laboring under orchitis presents himself to a surgeon, that he is immediately suspected of a blennorrhœa, and there is no credit given to his protestations that it has been produced by violent efforts. Nevertheless there is sometimes no trace of urethral discharge, nor any evidences of violence to the testicle; the epididymis is swollen, sensible and irregular; the tumefaction extends to the neighboring parts, and the patient cannot be made to confess any other cause than a violent effort.

It is now some years since, in examining the internal ring and the tissues concerned in its formation, we observed, as Thompson has asserted, that the right muscles give off many aponeurotic fibres which are attached to the internal face of the crest of the ilium. Those fibres form the inferior half of the orifice, and as a consequence pass under the testicular cord, or, to express ourselves in another way, and perhaps more clearly, the cord rides upon the fibres.

Now, keeping the disposition of the parts in mind, suppose the muscles to be in a state of contraction, those fibres must necessarily endeavor to straighten themselves, and if the effort is either considerable or long kept up, the cord must be compressed, and from this compression orchitis may ensue. Is it not highly probable then, that the urethra being in a state of irritation may predispose to orchitis; and a violent effort from the mechanism just pointed out produce it, in some cases at least?

Thus you will readily perceive that a man having a blennorrhœa is more disposed to contract orchitis than one whose urethra is in a state of health; but yet even in the absence of *ardor urinæ*, orchitis may occur under the influence of a violent muscular exertion. Formerly there was associated with swelling of the epididymis and testicle the idea of a metastasis; it is still the opinion of some, that the inflammation spreads from the urethra to the testicle suddenly and without any continuity of tissue.

This theory is overthrown by watching the moment that orchitis most generally makes its appearance. If it were by metastasis that the inflammation invaded the testicle, it would most naturally occur when the urethral inflammation was highest. Every one knows that precisely the contrary is the case; that orchitis is observed when the blennorrhœa is on the decline, and the patient nearly recovered. Inflammation of the testicle is very rare in the beginning of the disease.

Between the two classes of orchitis of which we have spoken there are yet other differences. The orchitis produced by external violence may terminate in various ways, but there has been observed in them a strong tendency to bring about suppuration, whilst blennorrhagial orchitis seldom terminates in that way. Other things being equal, traumatic orchitis in general is more grave than urethral orchitis, for it is evident that in producing suppuration the chances are that it will destroy the testicle. Blennorrhagic orchitis, on the contrary, only leaves behind it a little induration of the epididymis, and its functions are left unimpaired. Orchitis arising from external violence may leave behind it indurations and lumps of matter, which after a time may give rise to different kinds of tumors.

The inflammation of the testicle arising from a violent effort is either cured very speedily, or it requires more time to be dispersed than either of the others.

As for urethral orchitis it must not be thought that the sole cause of its origin is to be found in the existence of a blennorrhœa. It may be a consequence of divers injuries in the canal itself, stone, disease of the prostate and tubercles. Those cases of orchitis which arise from the last of these causes most generally end in tubercular disorganization of the testicle. The ordinary orchitis cannot be considered as a very grave affection, lasting only fifteen or twenty days under most circumstances.

The various methods of treatment adopted by surgeons are antiphlogistics, compression, and punctures made by the lancet. The first of these means may be employed, and if leeches are applied, I should advise them to be placed along the course of the cord rather than on the testicle itself. Compression has been revived by M. Friki; but it is a difficult method and is not free from danger. The testicle may be compressed too forcibly by the bandages, and unpleasant consequences ensue. The punctures by means of a lancet we are in the habit of using, and they are both inoffensive and efficient. If there be any collection of fluid it is evacuated, and you need have no fear about the little operation which is less difficult and dangeroas than that of bleeding; for what can there be to apprehend? There is no vessel in this region capable of furnishing a dangerous or embarrassing hemorrhage. Do you fear that you may reach the testicle? That is scarcely probable; and should it occur, it cannot produce the great inconveniences which were at one time apprehended. Under this treatment the ordinary sojourn of the patients in the hospital is seven days and a half.

We will proceed to examine the two methods of cauterization and scarification, and by comparing them with that of dilatation, we shall gain a clear idea of the relative advantages of these three methods.

We would say that cauterization does not succeed by itself; it must be assisted by dilatation. It is clear that if it act upon the narrowed portion of the canal by a destruction of substance it would be more dangerous than useful; for the retraction which would follow the healing of the wound would leave the passage still more restricted. We consider that there is a great error committed in supposing that nitrate of silver, introduced into the urethra, will destroy the tissues with which it is brought for so short a time in contact, for if you will examine the effect which follows on the skin from a similar cauterization, you will find that it is most superficial, that no eschar results from the touch, and we have every reason to believe that similar results occur in cauterization of the urethra. Therefore, by cauterization, thus employed, there is obtained, at best, but a mere modification of tissue, as is observed in some diseases of the skin, in certain cases of lichen, eczena, &c.; and if, concurrently with cauterization, dilatation were not employed, what power could it be supposed to exert upon the stricture? Here then, you see, is the reason why we employ only dilatation. If you have actually cauterized the urethra, you have performed a bad operation, because it is done at an expence of tissue; this is destroyed, and there will only follow a more decided stricture. If, on the other hand, only a slight modification of tissue is obtained, you have done no further good than dilatation would have effected had it been employed alone. Why, then, not trust to dilatation by itself? And, furthermore, we have not sufficiently thought, perhaps, that with bougies in the urethra a compression is produced which, of itself, acts upon the inflammation of the tissues. Among the other means employed in strictures, we may mention abrupt dilatation and scarifications. Abrupt dilatation is already nearly abandoned; it is understood to consist in passing a very large sound through a very small stricture. This is a most curious idea; nevertheless there is at the bottom of seemingly the merest paradoxes something which is true; and when such propositions are announced by men of such talent as M. Mayor, it cannot be doubted that their authors have witnessed some cases to which the method is applicable; these cases, we suspect, are those in which you detect small valvular openings. It has been advised to dilate the urethra by an instrument which, penetrating at first in a reduced form, is capable after being introduced, of being enlarged. We cannot give our sanction to this method, though some surgeons have assured us that it is a good one. Scarifications or incisions have been, by turns, too much praised, and too much undervalued. In ordinary cases we do not believe that they possess any advantages over dilatation; but when the cicatrix is hard, as in ligneous strictures, (*coarctation ligneuses,*) this is a method which is sometimes employed. It is established, however, that in thirty cases of stricture, you would not encounter more than three or four of this description. The partisans of scarification generalise the method, and believe it possesses advantages over other means; however, bear in mind that they associate with it dilatation. As for us, if we must sum up our opinion of the relative utility of each of the methods we have just spoken of, we should say, in the ordinary strictures from diffuse inflammation, and presenting a double cone, dilatation alone, or employed, if you wish, with cauterization by nitrate of silver, is the remedy; we reject caustic, potash, Vienna paste, &c.

In strictures which are very resisting, hard, woody, employ scarifications. In strictures consisting of thin bridles, and in valvular strictures, resort to the abrupt dilatation of M. Mayor. We look upon medicated bougies as entirely inefficient.

Such is an imperfect translation of a lecture of Professor Velpeau's, to which I listened with great interest, and which I hope will not be read without some satisfaction. I will conclude my present communication with a few items of medical and scientific news, which appear to me interesting.

M. Beneditti, an Italian physician, speaks very highly of the tannate of iron in chlorosis. He commences by administering nine grains, and increasing the dose gradually to thirty-six grains, in the twenty-four hours. In plethoric and very robust individuals he uses larger doses.

Dr. Guépratte has proposed a method for preparing the moxa which seems to possess some advantages over the ordinary processes. Instead of paper or carded cotton, he employs calico which has been freed from its starch. This he saturates with a solution of the sub acetate of lead, and dries and rolls it into cylinders of the requisite size. Upon the part where he designs applying the moxa he drops a very small quantity of a solution of gum Arabic, which assists to keep the moxa in its place. The moxa made in this way, he says, ignites readily and burns regularly, without odor, smoke, or flame. The operation is more prompt, less difficult, and less painful than when the moxa of carded cotton is used.

For a number of years Dr. Gower has been in the habit of employing, with great success, topical applications of the infusion and alcoholic tincture of tobacco in prosopalgia, and he was not sure whether it acted as an excitant or sedative, until M. Chippendale discovered that the active principle of this vegetable was nicotine. Dr. G., of all the various preparations of nicotiana, is most partial to the extract. He has seen three cases of this obstinate neuralgia yield instantly and permanently to a single application of the aqueous solution of this extract. And its success when rubbed upon the diseased jaw was no less marked in a case of tooth-ache.

La Verrier, who has immortalized himself by the discovery of a new planet, is but thirty-one years of age. He was born in Lower Normandy, a province which, within the last century, has given to physical science, besides the young astronomer just mentioned, the following illustrious names: Rouelle, Vauquelin, Fresnel, Dumont, d'Urville, and Laplace. The Minister of Public Instruction has just addressed a letter to the Academy of Sciences concerning the discovery of La Verrier, in which he says: "I think it right to inform the Academy of Sciences that the King has, in consideration of the event which now interests the scientific world, named M. La Verrier officer of the royal order of the Legion of Honor. He had not arrived at his turn by time; but science has its services of exception and its actions of eclat as well as war. The labors of M. La Verrier are of the class which honor our age and our country."

You have heard, of course, of the fulminating cotton, first prepared by Professor Shönbein, of the University of Bâle, which is creating a great noise all over the world. The discoverer has pretty clearly shown, in his various experiments, that the inflammability of this new explosive is greater than that of gunpowder; it has several times been placed upon gunpowder and exploded without igniting that substance. The combustion of the cotton creates no smoke, affords no appreciable odor, and leaves no residuum. Its immersion in water, provided it is afterwards carefully dried, does not in the slightest degree impair its explosive powers, and the cotton after it is prepared, retains its natural properties. Dr. Otto, of Brunswick, thus describes the process of preparing the cotton, which will be seen to be exceedingly simple. The cotton is to be steeped for half a minute in concentrated nitric acid, then taken out and immediately thrown into water, which is changed until all traces of the acid have disappeared. That which is in lumps or rolls must be carefully opened in order to free it more perfectly from the acid, after which it is thoroughly dried. The smallest quantity of the cotton thus prepared, placed upon an anvil and struck with a hammer, produces an explosion like the fulminating silver. When used in fire arms, it is made into a small roll and wadded down as is practised with gunpowder. Time will fix the value of this article as a substitute for powder, but if it is kindled with such facility by the blow of a hammer, will it not increase very seriously the danger of handling guns?

The percussion of the ramrod driven down with more than the gentlest force, would probably ignite the cotton to the great hazard of the sportsman. And, again, when the gun became heated by repeated discharges, the ignition of the material could hardly be avoided. For these reasons, I should doubt whether the explosive cotton can ever come into general use as a substitute for gunpowder.

M. Sanson, one of the *savans* of this great metropolis, has lately addressed to his government a long letter, in which he recommends the King to prohibit the importation of all *venomous reptiles* into France, except for the *Jardin des Plantes.* He draws a frightful picture of the disasters which might flow from the escape of a single female rattlesnake big with young. Her eggs, he fancies, would all hatch, and the little snakes all grow to be big ones, and in their turn go on producing new generations of snakes until France would be overrun with these formidable reptiles. Several of the papers ridicule the suggestion as very absurd. If a female snake should escape, say they, it would perish from the cold of the first winter; or if she should survive, the heat of the summer would not be sufficient to hatch her eggs. But are they sure that the rattlesnake lays eggs? I am sure it is stated in some works on herpetology that the venomous snakes bring forth their young alive, and not in the shape of eggs. Nevertheless, the fears of M. Sanson may well excite the merriment of his countrymen.

